# Using focus groups to evaluate a multiyear consumer health outreach collaboration

**DOI:** 10.5195/jmla.2021.987

**Published:** 2021-10-01

**Authors:** Tara Malone, Shari Clifton

**Affiliations:** 1 tara-malone@ouhsc.edu, Assistant Professor and Head of History of Medicine & Serials, Robert M. Bird Library, University of Oklahoma Health Sciences Center, Oklahoma City, OK; 2 shari-clifton@ouhsc.edu, Professor, Associate Director, and Head of Reference & Instructional Services, Robert M. Bird Library, University of Oklahoma Health Sciences Center, Oklahoma City, OK

**Keywords:** consumer health information, outreach, qualitative research

## Abstract

**Objectives:**

Academic health sciences librarians sought to evaluate the efficacy and future of the Health Information Specialists Program, a five-year consumer health information outreach collaboration with public libraries across the state.

**Methods:**

Five focus groups were held with participants from all five years of the program. Thirty-four participants from the program attended. Facilitators used structured interview guides consisting of eleven questions regarding the impact of the collaboration on participants' abilities to connect themselves or others to health information; the usefulness of materials or knowledge gained and its applications; any consumer health outreach projects that arose from the program; and suggestions for future topics, formats, or modifications. Data was hand-coded and analyzed using the framework analysis methodology for qualitative research.

**Results:**

Participants reported feeling improved confidence and comfort in providing health information services to their patrons. Numerous instances of knowledge transfer—in their personal lives, with their colleagues, and for their patrons—were described. Participants reported improved abilities to both find and evaluate consumer health information, and many adapted class materials for their own programming or teaching. Suggestions were provided for future class topics as well as a program website.

**Conclusion:**

Based on data from the five focus groups, the Health Information Specialists Program has positively impacted participants in a number of ways. Primary among these were self-reported improvement in both health information retrieval skills and the ability to evaluate the reliability of health information online, as well as in the confidence to help patrons with their health information needs.

## INTRODUCTION

The majority of US adults search for health information online, with most relying on search engines such as Google or Bing to find health-related content [1–3]. In this age of digital health information—and misinformation—public libraries have a crucial role to play in the health and health literacy of the communities they serve [4]. This includes ensuring that patrons have the skills to find and assess reliable consumer health information on the web. In the United States, nearly eight in ten adults state that public library staff help them find reliable information. Additionally, nearly 38% of library Internet users seek health information [5–6]. Public libraries are viewed as trustworthy arbiters of information and have long provided their patrons with health-related programming and health information resources, as well as other offerings that impact health literacy in their communities [7–8].

In their 2010 Action Plan to Improve Health Literacy, the US Department of Health and Human Services called upon public libraries and medical librarians to collaborate toward creating community health resources and to train more public library staff in the applications of health literacy skills and technologies in order to help patrons [8]. Patron needs are acute, and many library staff need additional training, resources, and partners in order to provide robust health information support [9]. Health sciences and public libraries have a long history of creating partnerships and are natural collaborators for addressing the health information needs of local communities [10–11]. To this end, reference librarians at the University of Oklahoma Health Sciences Center Robert M. Bird Library created the Health Information Specialists Program (HISP) for public library staff across the state of Oklahoma. The aim of this program was to create a network of health information specialists in public libraries through a series of in-person and online classes, webinars, and other events introducing public library staff to reliable online consumer health information resources. Each year of the program culminated in the achievement of Level 1 or 2 Consumer Health Information Specialization (CHIS) from the Medical Library Association (MLA) for participants with enough hours to qualify, the cost of which was covered by funding from the National Network of Libraries of Medicine, South Central Region [12]. Participants in Years 3–5 of the program also earned continuing education credits for the Public Librarian Certification Program managed by the Oklahoma Department of Libraries. (Note: While the HISP was designed for public library staff and the large majority of participants fell into this category, there was also participation from tribal, state, and academic librarians, as well as from literacy staff and library school students.)

Upon the conclusion of Year 5 of the program, 106 library staff from 67 Oklahoma libraries completed Level 1 or 2 CHIS. To analyze the impact of the first five years of the program and determine a path forward for future offerings, we performed a systematic assessment of attendees' health information experiences during and subsequent to their HISP participation. Only individuals who completed requirements for Level 1 or 2 CHIS through the program were eligible for the study. We specifically sought to determine:

1. How did participants feel the HISP impacted their ability to connect themselves or others with reliable consumer health information?
2. Have materials or knowledge gained from the program been utilized, adapted, or shared in a personal or professional capacity and, if so, in what manner?
3. Are participants undertaking health information outreach projects subsequent to their participation and, if so, what are these?
4. How can the program be modified to help participants expand knowledge of and access to reliable consumer health information?

## METHODS

**Figure 1:**
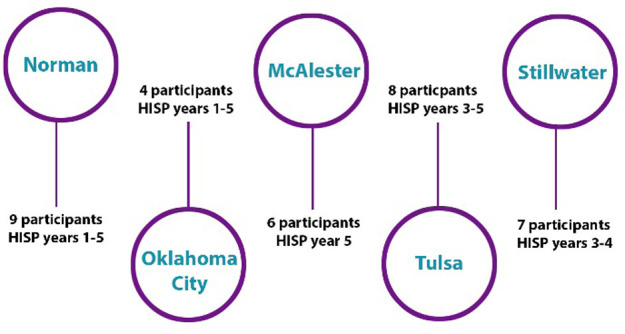
Study sites and samples

We were especially interested in understanding the qualitative “stories” behind each HISP participant's experience in the program. In order to capture a rich variety of data, we conducted a series of five focus groups lasting 2.5 hours each at sites across the state of Oklahoma (Figure 1). Per recommended best practices, each group was limited to no more than ten participants to better ensure that every group member could thoroughly contribute their perspectives [13]. Participants were recruited via phone or email from the roster of HISP trainees who achieved their Level 1 or 2 CHIS.

We served as facilitators for each session using a semistructured interview guide consisting of eleven questions (Table 1). Each focus group was divided into three discussion sections and was recorded using two portable digital audio recorders (in case of technological failure). Participants were required to sign an informed consent document and were offered $25 gift cards in appreciation of their time. The research protocol and all study materials were approved by the University of Oklahoma Health Sciences Center Institutional Review Board.

**Table 1:** Interview questions

**Discussion 1**
How (or has) your participation in the Health Information Specialists Program impacted how you search for health information? What were the most useful aspects of the Health Information Specialists Program for you either personally or professionally? What about the least useful aspects? Has earning your Medical Library Association Consumer Health Information Specialization and/or continuing education units through the Oklahoma Public Librarian Certification benefitted you in any way? Why or why not?
**Discussion 2**
Discovering how any knowledge or materials gained from the Health Information Specialists Program has been used to help others is very important to us. Please describe: Any ways you have utilized the knowledge, resources, materials, or relationships you gained from the Health Information Specialists Program to serve your patrons/customers, colleagues, family members, etc. Any other health information projects you are engaged in or are considering What barriers, if any, have you or someone you know encountered when applying information or exploring resources from the program? Please describe any collaborations that have arisen from your participation in or knowledge gained from the program, or collaborations you are considering.
**Discussion 3**
What are some health information topics you think would be important for future Health Information Specialists classes/events to address, and why? Are there events or formats in addition to in-person and online classes you would like to see in the program's future? Examples might include ready-to-adapt materials, development of a virtual community to share knowledge and experiences with other members of the program, etc. What particular populations of patrons/customers or areas of the state would you like to see specifically addressed through future Health Information Specialists Program offerings? Examples might include persons with disabilities, rural citizens, patrons/customers in poverty, or minority groups. What are some potential strategies we should consider to expand the Health Information Specialists Program to other information professionals and community partners in Oklahoma? Examples might include tribal librarians, literacy coordinators, local health and wellness organizations, etc. In general, how could we improve the program in the future?

To efficiently organize and analyze the large volume of data, we used the framework analysis methodology as described by Gale et al. (Table 2) [14]. This method of analysis, while infrequently appearing in library research, is commonly used for qualitative studies in health care fields. We selected this seven-step methodology based on several factors, including its flexibility in allowing both deductive and inductive analysis; the charting procedure, which helped streamline and manage the large volume of data; and the overall systematic nature of the method, which allowed a rigorous approach to qualitative data without sacrificing its integrity and potential. We used the original research questions as deductive prompts for data coding in coordination with open coding for inductive analysis.

**Table 2:** Steps of the framework analysis

Step 1: Transcription	Recordings were sent to a professional transcription service approved by the institution, which generated approximately 450 pages of transcripts Transcripts were double-checked and corrected by the investigators, at which point all recordings were destroyed All transcripts were de-identified and participants were instead assigned a number
Step 2: Familiarization with the interview	Investigators listened to recordings multiple times during quality assurance of the transcription service Transcripts were read thoroughly at several points throughout the analysis phase
Step 3: Coding	Each investigator individually applied an initial round of both deductive and open coding to one site transcript, then met to combine initial codes into the first iteration of the analytical framework Each investigator coded by hand
Step 4: Develop a working analytical framework	A set of categories, codes, and definitions was created by investigators based on initial coding of the first transcript This document constituted the first iteration of the analytical framework (Figure 2)
Step 5: Apply the analytical framework	The framework was then applied to the initial transcript again and to subsequent transcripts thereafter Adjustments to the framework were made as needed throughout the process upon regular discussions between investigators
Step 6: Chart data in to the framework matrix	Data was charted into the framework matrix to organize and streamline analysis Although qualitative analysis software such as NVivo is capable of automatically generating a framework matrix based on the data, investigators felt that creating their own matrix helped assure quality and emphasize familiarity with the data
Step 7: Interpret the data	The final framework matrix was consulted and used to identify themes and patterns in the data.

**Figure 2:**
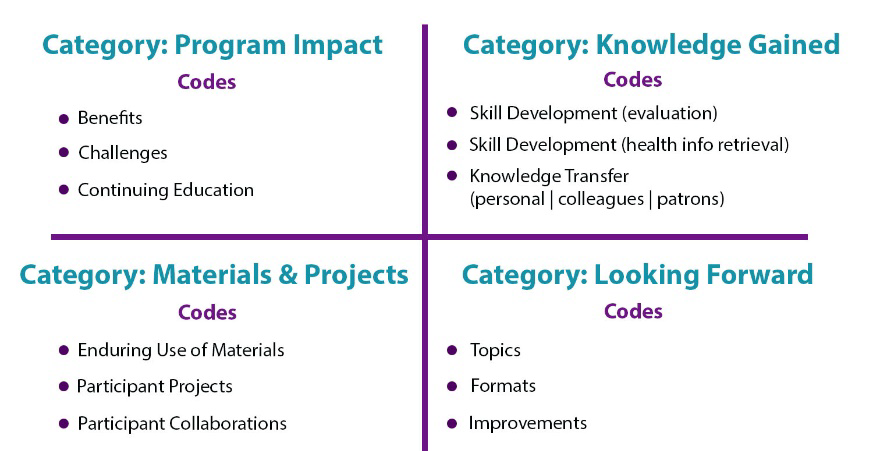
Examples from the analytical framework

Perhaps one of the most important steps in applying the framework analysis methodology came in the iterative development of the analytical framework. This document was revised multiple times as reviewers coded data, compared, and discussed, then revised the analytical framework and applied it again to the data. Examples are provided in Figure 2.

## RESULTS

### Research question 1: Program impacts

The most commonly described benefit of HISP participation for public library staff was increased confidence in finding and delivering consumer health information. This was particularly true in the area of health information interactions with patrons. Participants repeatedly expressed that with the information gained, they felt more competent and comfortable providing health information to others. Much of this increased self-assurance was directly credited to an improved awareness of reliable online consumer health information resources introduced by the program. One participant said, “It just gave me confidence in knowing which websites to trust.” Another said, “I think I'm much more aware now of how much free information and how many free resources are available to consumers.”

Participants also felt that attending the program—and particularly earning their Level 1 or 2 CHIS upon completion—increased their credibility in the eyes of patrons, supervisors, and even funders. They described how mentioning that they had such expertise as the CHIS often helped smooth potentially sensitive interactions with patrons who were seeking health information. Some participants mentioned using the information on their résumés, and one manager described how beneficial it was to their library system that employees had earned this specialization. Additionally, some participants stated that when applying for funding, earning the CHIS lent them some positive leverage toward developing competitive applications.

Many participants also emphasized the rewards reaped from serendipitous knowledge transfer during the program. Several indicated that the interactions with participants from different libraries—particularly during in-class, in-person discussions prompted by instructor-led activities—was a direct benefit of the program.

### Research question 2: Knowledge gained

According to King, knowledge transfers “are between a clearly defined source and a recipient, have a focus, and have a clearly identified objective” [15]. In the case of the HISP program, initial knowledge transfer regarding health information resources occurred unilaterally from program instructors to participants. Following completion of the program, knowledge transfer then fell into three categories: personal (participants drew upon their newly gained knowledge and applied it to their own personal health information needs or those of family or friends); patrons (participants used knowledge gained from the program to assist patrons with health information inquiries); and colleagues (participants shared program knowledge gained and utilized resources to assist colleagues with health information needs). In some cases, examples that encompassed multiple types of knowledge transfer were shared. Regarding the foundational class in the program, one participant said, “I think one of the most useful aspects is that first class about how to explore research and stuff, just because you can teach that relatively quickly to patrons and feel like you are teaching them health literacy – the very basics so then we can go on to maybe more specifically their thing. And even just getting that one [class] to as many library staff as possible feels like a big impact.” Table 3 offers examples of knowledge transfer in each category communicated during the HISP focus groups.

**Table 3:** Knowledge transfers subsequent to HISP participation

Type of knowledge transfer	Transfer example
Personal	Recognizing pharmacy-issued erroneous medication using resources discovered in HISP classes Using class resources to help an ill family member develop a list of questions to ask his health care provider regarding his condition Sharing resources with family members when a loved one was diagnosed with cancer Identifying pills left in the library using class tools
Patrons	Providing lists of online health information resources from HISP classes to patrons Helping patrons connect to health-related community organizations and resources Assisting patrons in finding reliable online information after diagnoses Offering one-on-one health information appointments for patrons
Colleagues	Adapting HISP courses to hold system-wide trainings Sharing the impact of the HISP at a poster at the Public Library Association annual meeting Providing a chronically ill colleague with online resources from program classes Directing colleagues to National Library of Medicine online resources for their personal use or for the development of consumer health programs

In addition to transfers of knowledge, participants in all focus group sessions communicated improved skills in health information retrieval, as well as the ability to better evaluate the credibility of online health information resources. Many indicated that prior to participation in the HISP, they had almost exclusively relied upon search engines to find health information for patrons and themselves. One participant said, “I was one of those people that just automatically went to Google and just clicked the first thing I saw, and went from there.” Armed with knowledge from the program, they felt better able to 1) have a starting place other than a search engine for online health information inquiries and 2) be more confident in selecting reliable health information results from online searches.

### Research question 3: Materials and projects

Each participant in the HISP received a personal binder of class materials, resource lists, and additional consumer health information resources. Attending several classes in the program and building the binder resulted in a comprehensive reference tool for many consumer health topics. During the focus groups, multiple participants reported using the information in the reference binder frequently, both for their own personal use and for their patrons. Several reported keeping their binder at their library's reference desk for ready consumer health information if needed. Additionally, during the last three years of the HISP, a webpage listing resources by class name for the respective year was provided to class attendees. Participants relied on each site for a convenient list of consumer health information links and also shared it with patrons and colleagues.

Focus group members reported regularly adapting materials and resources from the classes for their own teaching and programs. Participants from several libraries adapted HISP materials to offer “train-the-trainer” sessions to other colleagues within their system. One participant applied for funding to adapt and teach a HISP course for staff at different small libraries across the state and used program materials to develop a resource-focused website. They said, “They just loved it, these librarians at these small libraries learning how to use these resources and really being amazed and happy about these resources on the library website. It's been so helpful to me to borrow what you have done.” For patrons, participants used HISP materials to provide training sessions and adapted materials for health fairs. They also created displays and “resource centers” of materials adapted from the program and taken from program resources. One group of library staff used adapted HISP materials to supplement programming for a healthy living grant project. Other participants received a grant to teach part of the HISP series while providing community members with materials, resources, and access to online resources from classes; incorporating class materials and resources into computer training opportunities for patrons; and making computers with class resources available to members of the public.

### Research question 4: Looking forward

Participants in each group expressed a desire for a single website for the program. Ideally, participants wanted a site that would combine elements from all years of the HISP, including resource lists, ready-to-adapt materials such as brochures and bookmarks, and information from the class binders such as exercise questions and other class materials. In addition, they expressed a desire for the site to serve a social collaboration function, with members of the HISP cohort able to post and share regarding their experiences using knowledge gained from the program. This desire almost exclusively constituted the feedback regarding program improvement. A small number of participants were specifically in favor of more ready-to-distribute materials, which could be available on a website. For example, one participant asked for resource bookmarks. “It's just something easy we can quickly give them [patrons],” they said. “Something like that would be a great tool.” Others expressed that brochures serving similar functions would be a good addition to the program. Other participants requested continued in-person training, stating, “Just being able to have that interaction makes it that much more memorable.”

Participants were asked which topics they most desired to see covered in future HISP offerings. The most popular subjects included pet health, mental health, and healthy aging. While the latter two topics have been the subject of HISP classes before, they have not been consistently offered every year.

## DISCUSSION

Public libraries are a natural fit as contact points for health information, offering access to authoritative resources, healthy living activities, and health care and insurance programming [16]. The findings of these focus groups dovetail with the growing recognition of the importance of public-academic or public-medical library partnerships to promote consumer health, as demonstrated by such national initiatives as Promoting Healthy Communities and the awareness campaign for the All of Us Research Program (both coordinated by the Public Library Association and National Network of Libraries of Medicine), as well as by examples of numerous smaller training partnerships happening day-to-day [17, 18].

However, while awareness of the potential of these partnerships is on the rise, obstacles remain. In a time during which they are increasingly called upon to answer health-related queries, many public library staff do not feel adequately trained to assist patrons in need of health resources or information [19, 20]. Based on direct feedback from participants, the HISP addresses this need by providing a training program that results not only in an increased awareness of resources, but also a much-needed boost of self-assurance for library staff meeting the health information needs of patrons. In addition to a crisis of confidence on the part of library staff, while the number of library partnerships may be growing, the impact of these programs often goes unknown because while post-training surveys occasionally occur, longitudinal follow up is either not conducted or not often published. As such, while there is a growing number of examples of public-academic-medical library collaborations regarding consumer health, few published studies have evaluated the extended impact of their programs and any accompanying training for library staff. Based on the results of this study, a potential strength of the HISP program is its already demonstrated potential to self-sustain. Participants from a variety of timeframes have shared experiences of using HISP materials and classes to pay the information forward, both with patrons and with library colleagues. In addition to future offerings, projects such as the upcoming program website will foster the capability to design further projects using HISP resources as well as maintain the networking that has been a strength of the program.

While this study indicates that the HISP clearly addresses the needs of its participants, the study itself was not without challenges and limitations. As with any qualitative research, the amount of data generated from the focus group transcripts was vast. The processes to secure transcription, deidentify participants, and code and chart data were all especially time consuming. Even though professional transcription services were secured, this alone was challenging, as transcriptionists had no context for the project and transcript correction was a lengthy process to ensure accuracy. Frequent meetings between investigators to refine the analytical framework, as well as to compare coding and interpretation consistency, were lengthy but necessary for a project of this magnitude.

There are pros and cons to investigators also serving as moderators for focus groups, and this study is no exception. On the one hand, it is important for moderators to have a healthy context for and understanding of the focus group topic [13], thus making principal investigators seem like a natural fit as moderators. However, inserting investigators into the focus group also can introduce bias into the procedures and results. When faced with the choice, we decided that due to logistical limitations, we would also serve as moderators for this study. However, this is a potential limitation that should be noted. Finally, the HISP has evolved greatly over the past several years and has differed between year cohorts. It is possible that because some focus groups did not represent all years of participation, data could be biased toward participants' respective years.

Based on data from the five focus groups, the HISP has positively impacted participants in a number of ways. Primary among these were self-reported improvement in both health information retrieval skills and the ability to evaluate the reliability of health information online, as well as in the confidence to help patrons with their health information needs. An important component of the HISP was the ability to earn MLA's Consumer Health Information Specialization, which provided participants with additional credibility with patrons, supervisors, and funding organizations.

In addition to applying knowledge gained from the program to their own personal health information needs, participants frequently shared and adapted materials, resources, and other information gained with patrons and colleagues. This includes both print materials provided during each class and resource lists from class webpages.

Participants as a whole indicated their interest in future HISP offerings, especially regarding either new topics or topics that were not taught in their respective year(s). Additionally, there was an emphatic desire for a unified web platform for the program. Subsequent to the study, we secured funding from the National Network of Libraries of Medicine South Central Region to develop requested courses and build a HISP website, and a beta is approaching launch. Additionally, classes are being developed according to the topic requests of participants.

## Data Availability

Permission to share the data from this study was not provided by our institution's IRB or by participants, therefore it cannot be made available at this time.
